# Reply to Record *et al.* “The role of *PMP22* T118M in Charcot–Marie–Tooth disease remains unsolved”

**DOI:** 10.1016/j.jbc.2023.105181

**Published:** 2023-09-14

**Authors:** Katherine M. Stefanski, Geoffrey C. Li, Justin T. Marinko, Bruce D. Carter, David C. Samuels, Charles R. Sanders

**Affiliations:** 1 Department of Biochemistry, Vanderbilt University School of Medicine, Nashville, Tennessee, USA; 2 Center for Structural Biology, Vanderbilt University School of Medicine, Nashville, Tennessee, USA; 3 Department of Molecular Physiology and Biophysics, Vanderbilt University School of Medicine, Nashville, Tennessee, USA; 4 Department of Medicine, Vanderbilt University School of Medicine, Nashville, Tennessee, USA

*PMP22* encodes peripheral myelin protein 22. We agree that T118M *PMP22* is not the cause of a Mendelian disease under WT/T118M heterozygous conditions—this is a major conclusion of this article (1). However, our data are consistent with previous reports of linkage of T118M *PMP22* to very rare cases of mild Charcot–Marie–Tooth (CMT) disease. Record *et al.* are in agreement with our statement that the penetrance of T118M is <5% but make the valid point that a more thoughtful analysis sets the upper limit of penetrance to an even lower percentage of heterozygous carriers (<1.2%).

Data for WT and T118M PMP22 surface trafficking efficiencies as a function of total expression level are presented in Figure 3 of Ref. (1). Corresponding data for other CMT disease mutations (mild, moderate, and severe) as a function of cellular expression data were previously reported (Fig. 3 in Ref. (2)). There, two PMP22 variants known to cause mild or moderate forms of CMT exhibited WT-like behavior in that their cell surface trafficking efficiencies go down as total expression levels are increased—different from what was reported in this article for T118M. For L16P (severe disease), trafficking and total expression are uncoupled at all but the lowest levels of expression, like T118M. This led us to look at aggregation to uncover further differences between L16P and T118M, as described in this work (1).

The database used to establish a relationship between T118M PMP22 and the likelihood of suffering from repeated episodes of carpal tunnel syndrome contained 88,308 records. Carpal tunnel syndrome was chosen for the analysis since it has a much larger population frequency than does CMT. Given that CMT is a 1:2500 disease, we expected on the order of only 35 CMT patients to be represented in those records. With the very low penetrance of the T118M PMP22 mutation in CMT, this means that we would expect to observe few if any T118M PMP22 carriers among these ca. 35 cases. In light of this consideration and because the available method (manual) for sorting through the electronic patient records was very laborious, we did not undertake the analysis Record *et al.* would have preferred to have seen.

Finally, we note that this work was conducted in light of literature controversy as to whether T118M *PMP22* is a CMT disease variant in WT/T118M heterozygous humans. Our studies offer a model that potentially resolves this controversy by positing that T118M *PMP22* seems to be only a very low penetrance risk factor for CMT. We also suggest why. While the T118M PMP22 protein resembles 100%-penetrant PMP22 variants in that it is unstable (3), prone to mistraffic (1, 3, 4), and exhibits reduced partitioning into ordered-phase membrane domains (5), this work (1) shows that the T118M protein differs from other disease variants and even from WT in that it is resistant to formation of intracellular aggregates.

## Conflict of interest

The authors declare that they have no conflicts of interest with the contents of this article.
